# The Utility of Novel pH-Impedance Monitoring Parameters (PSPW Index and MNBI) in Pediatric Gastroesophageal Reflux Disease Phenotypes—A Systematic Review

**DOI:** 10.3390/jcm13113351

**Published:** 2024-06-06

**Authors:** Radu Samuel Pop, Dorin Farcău, Lăcrămioara Eliza Chiperi, Dan Lucian Dumitrașcu

**Affiliations:** 1 3rd Department of Pediatrics, “Iuliu Hațieganu” University of Medicine and Pharmacy, 400217 Cluj-Napoca, Romania; 2 3rd Pediatric Clinic, Clinical Emergency Hospital for Children, 400217 Cluj-Napoca, Romania; dorin.farcau@umfcluj.ro; 3 Department of Pediatrics, George Emil Palade University of Medicine, Pharmacy, Sciences and Technology, 540136 Târgu Mureș, Romania; lacramioara-eliza.pop@umfst.ro; 4 2nd Department of Internal Medicine, “Iuliu Hațieganu” University of Medicine and Pharmacy, 400006 Cluj-Napoca, Romania; ddumitrascu@umfcluj.ro

**Keywords:** GERD, PSPW, MNBI, pediatric

## Abstract

**Background/Objectives**: Researchers have proposed two novel impedance-pH parameters, mean nocturnal baseline impedance (MNBI) and the post-reflux swallow-induced peristaltic wave (PSPW) index, to enhance the diagnosis of gastroesophageal reflux disease (GERD) and enable better predictions of the effectiveness of anti-reflux therapies. This systematic review aims to synthesize the available evidence on the utility of the PSPW index and MNBI as diagnostic tools for pediatric GERD. **Methods**: A systematic search of studies reporting PSPW index and MNBI values in patients with GERD was performed in PubMed, Embase, Clarivate, Scopus, Cochrane and Google Scholar databases from their beginning until April 2024. The following terms were used: *GERD*, *children*, *pediatric*, *PSPW* and *MNBI*. **Results**: Eight studies were included, describing 479 patients ranging from 2 months to 17 years old over an 8-year period in 12 pediatric centers. Four studies demonstrated that children with pathological acid exposure have a significantly lower MNBI, with a good discriminatory ability to diagnose GERD. The PSPW index showed lower values in patients with reflux hypersensitivity (RH) compared to those with functional heartburn (FH). **Conclusions**: Patients with pathological acid exposure tend to exhibit lower MNBI and PSPW index values compared to those with normal acid exposure. MNBI and the PSPW index show promise as diagnostic tools in distinguishing between different GERD phenotypes. Further research is needed to establish standardized diagnostic criteria and optimize the clinical applicability in GERD diagnosis and management.

## 1. Introduction

Gastroesophageal reflux disease (GERD) represents a prevalent gastrointestinal disorder in pediatric populations and is characterized by the abnormal reflux of gastric content into the esophagus, leading to a spectrum of symptoms and potential complications [1,2,3]. Conventional diagnostic methods for pediatric GERD, such as pH monitoring and endoscopy, have inherent limitations, particularly in capturing non-acid reflux events and assessing the reflux phenotype [4,5].

Multichannel intraluminal impedance-pH (MII-pH) monitoring is currently established as the foremost diagnostic modality for detecting gastroesophageal reflux (GER), as it enables the quantification and description of all reflux events and their potential association with symptoms, delineating several GERD phenotypes, as categorized in the Rome IV esophageal criteria. Non-erosive reflux disease (NERD) is a condition in which patients experience GERD symptoms and have no esophagitis on endoscopy but abnormal acid exposure during MII-pH monitoring. Reflux hypersensitivity (RH) is characterized by a negative endoscopy and normal acid exposure of the esophagus but a positive association between symptoms and reflux episodes. Functional heartburn (FH), on the other hand, is associated with the typical GERD symptoms but a negative endoscopy, normal esophageal acid exposure, and no association between symptoms and reflux episodes during MII-pH monitoring [6,7,8,9]. 

Researchers have proposed two novel impedance-pH parameters: mean nocturnal baseline impedance (MNBI) and the post-reflux swallow-induced peristaltic wave (PSPW) index [10]. These metrics are believed to provide a more precise evaluation of the GERD phenotype [11], enhance the diagnostic process [12,13], and enable better predictions of the effectiveness of anti-reflux therapies [14]. 

PSPW, characterized by an antegrade esophageal contraction following reflux events, reflects the effectiveness of the esophageal peristaltic response in clearing refluxed material from the esophagus [12]. MNBI, on the other hand, provides a measure of the baseline electrical conductivity of the esophageal mucosa during periods with no reflux episodes, serving as a surrogate marker for mucosal integrity and reflux burden [15,16].

Studies in adult patients have shown that MNBI correlates with symptom outcomes, especially when the acid exposure time is inconclusive, indicating its utility in cases where traditional metrics fall short [17]. Additionally, MNBI has been proposed as a marker for laryngopharyngeal reflux, further expanding its diagnostic applications [18]. The PSPW index is crucial for evaluating esophageal chemical clearance and mucosal integrity, providing a comprehensive assessment of GERD [19]. These novel impedance parameters have been found to increase the diagnostic yield of impedance-pH monitoring, differentiating between NERD, RH, and FH [12,15,20]. 

Furthermore, MNBI and the PSPW index have been suggested to be valuable in identifying proton pump inhibitor-refractory reflux disease, highlighting their importance in challenging cases [21,22]. These parameters have also been shown to link proton pump inhibitor (PPI)-responsive heartburn to reflux better than traditional metrics like the acid exposure time, emphasizing their superiority in certain diagnostic scenarios [21]. Overall, the inclusion of MNBI and the PSPW index in impedance monitoring protocols has significantly improved the diagnostic capabilities available for GERD, providing clinicians with valuable tools to enhance patient care and treatment outcomes [23,24].

Despite their promise, the utility of the PSPW index and MNBI in pediatric GERD remains incompletely understood. While studies in adult populations have demonstrated their potential diagnostic value, the pediatric population presents unique challenges and considerations that may influence the applicability and interpretation of these metrics [25]. As such, a comprehensive evaluation of the existing literature is warranted to assess the evidence supporting the use of the PSPW index and MNBI in diagnosing GERD in children.

This systematic review aims to synthesize the available evidence on the utility of the PSPW index and MNBI as diagnostic tools for pediatric GERD. By critically appraising the existing literature and identifying gaps in the knowledge, this review will reveal the current state of evidence and highlight areas for future investigation in pediatric GERD diagnosis and management.

## 2. Materials and Methods

An extended search was conducted using the standard methodology published by the Cochrane Collaboration [26,27] in multiple databases, including PubMed, Embase, Clarivate, Scopus, Cochrane, and Google Scholar, from their inception until April 2024. The following terms were used: *GERD*, *children*, *pediatric, PSPW* and *MNBI.* The review was not registered. The findings were documented in alignment with the guidelines provided by the Preferred Reporting Items for Systematic Reviews and Meta-Analyses (PRISMA) [28].

We included observational studies (retrospective and prospective) that evaluated the gastroesophageal reflux disease spectrum in which PSPW and MNBI were measured. We reviewed studies that involved populations consisting of neonates, infants, children, and adolescents. Studies that included pediatric patients with GERD but did not evaluate them through esophageal pH-impedance monitoring were excluded. Animal studies were also eliminated.

Two reviewers were used for the research. The interrater agreement reached 97% when identifying articles for inclusion in the review. In cases where the raters disagreed on a particular article, that article was excluded from consideration.

Patient characteristics such as age, the gastroesophageal reflux disease spectrum, endoscopic and histologic diagnosis, the duration of impedance monitoring, the type of novel impedance parameter measured, the statistical conclusion, and the clinical results were extracted from each publication.

To synthesize the data, we utilized Microsoft Excel version 2019. The results are presented as either mean with standard deviation or median with interquartile range, depending on the data provided in each article.

## 3. Results

**Figure 1:**
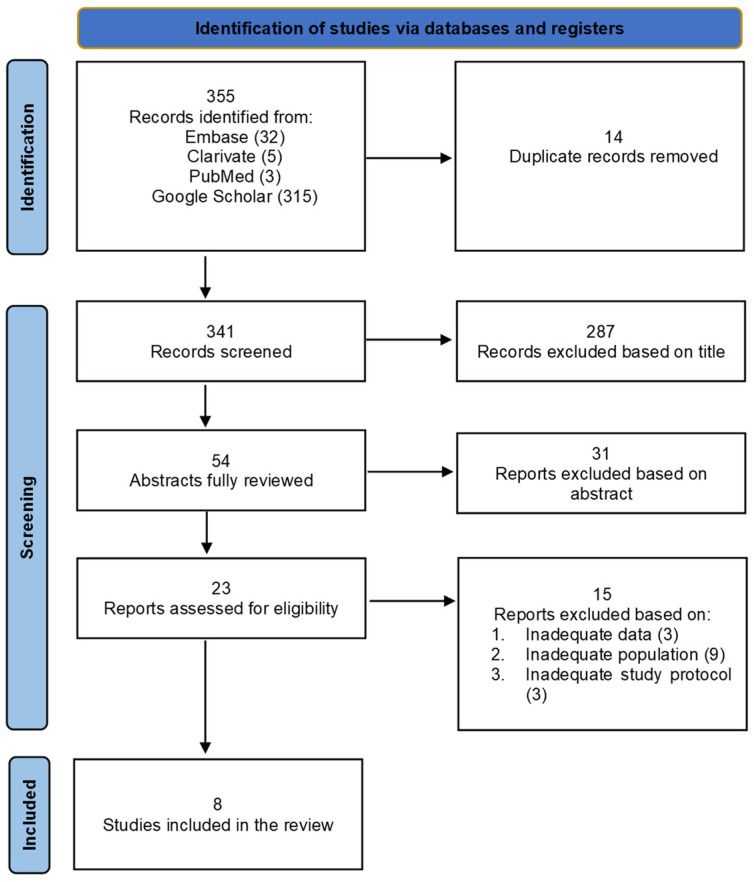
PRISMA 2020 flow diagram for new systematic review: study selection process.

The systematic literature search identified 355 publications, which underwent thorough screening based on their titles and abstracts. Following the removal of non-relevant articles and duplicates, 23 studies were deemed pertinent and evaluated. Ultimately, eight suitable articles were included in this systematic literature review. Details of the selection process can be found in Figure 1.

In the studies analyzed, a cohort of 479 patients ranging from 2 months to 17 years old were assessed over an 8-year period, from 2014 until 2022. The studies were conducted in a total of 12 pediatric centers across four continents. Patients with a diverse clinical spectrum of gastroesophageal reflux phenotypes were included, covering suspected GERD, diagnosed GERD, NERD, RH, and FH, as shown in Table 1. Table 2 summarizes the characteristics of the included studies. In the majority of studies, endoscopic and histologic confirmation of the GERD spectrum was established. Patients were off treatment at the time of impedance monitoring in all studies.

The types of impedance parameters that were determined were the PSPW index in six out of eight studies and MNBI in eight out of eight studies. In the majority of studies, data were reported as mean ± standard deviation, so an overall mean ± standard derivation was calculated for the principal gastroesophageal reflux disease phenotypes (GERD, NERD, RH, and FH), as can be seen in Table 3. One study [29] reported the impedance parameters as the median and interquartile rate, so its results were reported exactly and not included in the overall score. 

## 4. Discussion

Emerging evidence indicates the existence of different phenotypes in GERD, underscored by multifaceted underlying mechanisms, contributing to varying symptom perception and potential treatment outcomes [36]. Non-erosive reflux disease stands as the predominant type of GERD across all age groups, delineated recently into three distinct phenotypes based on the Rome IV esophageal criteria: NERD, RH, and FH, [1,24,37]. Blasi et al. [29] suggest that FH is the most prevalent pediatric non-erosive esophageal phenotype at 38.2%, followed by NERD at 26.5% and RH at 20.6%, confirming the accuracy of the existing data [37]. 

Understanding the mechanism implicated in the spectrum of GERD phenotypes in children is essential to tailor the treatment. 

Acid exposure of the esophageal mucosa is known to disrupt intercellular junctional complexes, leading to a subsequent leak between cells and the dilation of intercellular spaces in the epithelium. This damaged epithelium exhibits lower electrical resistance compared to healthy tissue, resulting in decreased impedance in pH-MII [38,39,40,41,42]. 

### 4.1. MNBI

Four studies included in this review [29,30,31,35] demonstrated that patients with pathological acid exposure have significantly lower MNBI values compared to those with normal acid exposure. This inverse correlation between acid exposure and MNBI has also been observed in adults [16,17,19]. 

Eiamkulbutr et al. [25] described MNBI as having a good discriminatory ability to diagnose GERD, with an area under the curve (AUC) of 0.726 (95% CI: 0.581–0.870). The study identified a cutoff value of 1466 ohms for MNBI, yielding a sensitivity of 50.0% and a specificity of 33.33%. In adult studies, MNBI was used to diagnose GERD, with an AUC of 0.876 (95% CI: 0.833–0.918). The best cutoff value identified was 2292 ohms [12]. Another study [13] reported a cutoff of 2061 ohms, with an AUC of 0.792. This cutoff yielded a sensitivity of 74.9% and a specificity of 67.4% [13]. Further research and validation are necessary to establish standardized diagnostic criteria and optimize the clinical utility of MNBI in GERD diagnosis.

Rosado-Arias et al. [33] showed that children presenting severe esophagitis have a lower MNBI compared to patients with non-severe esophagitis, suggesting that the measurement of nocturnal baseline impedance may serve as a valuable diagnostic tool, potentially obviating the necessity for upper gastrointestinal endoscopy in the assessment of esophagitis. Blasi et al. [29] demonstrated a reduced MNBI among children diagnosed with NERD compared to those diagnosed with RH and FH. In addition, Tortoriello et al. [30] revealed a significantly lower MNBI in pediatric patients with GERD compared to those with RH and FH, and Sabban et al. [32] showed statistically significantly lower values in children with RH compared to the group diagnosed with FH (*p* = 0.001).

These observations are consistent with the results described in adults, where MNBI has been shown to be useful in assessing GERD phenotypes accurately, especially when conventional metrics provide ambiguous results [11,22,43,44,45]. Studies have demonstrated that a low MNBI is associated with an abnormal reflux burden, fragmented peristalsis, and ineffective esophageal motility, such as erosive esophagitis; this is compared to those with less severe mucosal damage, such as non-erosive reflux disease and FH [12,45]. Additionally, MNBI has been shown to be lower in patients with RH and NERD compared to those with FH and healthy individuals [46]. Furthermore, MNBI has been found to distinguish patients with NERD from patients with FH with high accuracy [22,47].

In children, low MNBI values may serve as a useful predictor of endoscopically confirmed reflux esophagitis, offering the advantage of being less invasive and not requiring fasting or sedation. 

### 4.2. PSPW

Two studies [30,35] showed that the PSPW index was significantly lower in children with a conclusive GERD diagnosis compared to non-erosive phenotypes, revealing an inverse association between the PSPW index and acid exposure time. Blasi et al. [29] observed that the PSPW index was lower in NERD children compared to other non-erosive phenotypes. While no statistically significant variances were identified among various non-erosive reflux phenotypes, their study unveiled an inverse correlation between the PSPW index and acid exposure time, indicating a potential link between a lower PSPW index and a prolonged exposure to acid. 

In a study conducted by Sabban et al. [32], the PSPW index showed statistically significant lower values in patients with the RH phenotype compared to those with FH (*p* = 0.01). The same group conducted a two-center cohort study [34] that revealed differences in the PSPW index in children according to their clinical presentation. Those with the respiratory symptoms of GERD had a lower PSPW index and a higher MNBI compared to those with gastrointestinal symptoms. 

These results are consistent with adult studies that have strong data supporting the use of the PSPW index in investigating GERD patients. The PSPW index has been found to be lower in acid reflux-predominant GERD phenotypes, such as erosive esophagitis (EE) and non-erosive reflux disease (NERD), compared to esophageal hypersensitivity-predominant phenotypes [48]. 

A retrospective analysis involving adult GERD patients resistant to PPI treatment showed significantly lower PSPW index values in those with esophagitis who did not respond to treatment compared to those with healed esophagitis or non-erosive reflux disease (*p* = 0.003). This suggests that the PSPW index is a good measure of the effectiveness of clearing both acidic and weakly acidic refluxes [49]. 

Another study highlighted a strong negative relationship (r = −0.889) between the bolus clearance time and PSPW index, emphasizing the importance of esophageal clearance mechanisms [50]. Moreover, there was a positive correlation (r = 0.623) between the PSPW index and baseline impedance values, indicating the role of chemical clearance in maintaining mucosal integrity. The PSPW index also correlated directly (r = 0.626) with the esophageal contractile reserve, which inversely correlates with the acid exposure time, suggesting that acid exposure has an impact on esophageal muscle contractility [51]. 

In a prospective multi-center study [52], among the MII-pH parameters, only the PSPW index independently predicted resistance to GERD treatment (OR 1.082, 95% CI 1.022–1.146, *p* = 0.007). 

Furthermore, the diagnostic value of the PSPW index has been highlighted in adult studies evaluating its performance in identifying patients with reflux disease, with high AUC values observed, indicating its potential ability to perform accurate disease identification [11,12,20,45].

Limitations: The constraints of this systematic review stem from the limitations inherent in the studies that were included. Overall, a small number of patients, around four hundred (479), were included in the analysis due to the fact that some studies had small samples of cases (defined as fewer than 50 patients). Different definitions were used for pathological AET. Some studies used the British Society of Pediatric Gastroenterology, Hepatology and Nutrition BSPGHAN Motility Working Group criteria [53] (>7% in children aged ≥1 year, and >10% in children aged <1 year, or if reflux episodes occur ≥70 times in children aged ≥1 year, and ≥100 times in children aged <1 year or positive symptoms). Other studies [31] considered the pathologic AET as >5% in patients >1 year or >10% in those <1 year. Extended follow-up periods are necessary to enhance the assessment of the significance of these innovative parameters. Only one study [29] followed the patients in the long term (follow-up duration: 28.8 ± 21.8 months).

In summary, the studies reviewed provide valuable insights into the utility of the PSPW index and MNBI in children with GERD. They underscore the potential use of these parameters as diagnostic markers and emphasize the need for tailored approaches in pediatric GERD management. However, further research with a high number of patients is warranted to establish standardized protocols and guidelines for the use of the novel pH-impedance parameters in pediatric populations.

## Figures and Tables

**Table 1:** The clinical spectrum of the gastroesophageal reflux phenotypes in the included articles and the impedance parameters (PSPW index, MNBI).

Author, Publication Year	GERD Spectrum	PSPW Index %	MNBI (ohms)
Blasi, 2023 [29] (median, IQR)	NERD	42.6 (29.6–45.8)	1315 (1018–2832)
	RH	56.3 (38.7–67.2)	2724 (2273–3403)
	FH	52 (35.9–69.1)	2576 (2115–3014)
Eiamkulbutr, 2023 [25] (mean, SD)	extraesophageal GERD	56.11 (15.70)	1300.48 (600.31)
	no extraesophageal GERD	53.57 (27.59)	1897.69 (806.89)
Tortoriello, 2023 [30] (mean, SD)	GERD	20.11 (28.84)	1673.11 (598.67)
	RH	47 (29.23)	2303 (837.8)
	FH	52 (43.85)	2417 (656.23)
Rosado-Arias, 2022 [31]	pathological AET		2195
	normal AET		1997
Sabban, 2021 [32] (mean, SD)	RH	48.4 ± 20.84	1286 ± 514.68
	FH	64.6 ± 38.84	2168 ± 449.03
Rosado-Arias, 2021 [33] (mean, SD)	severe esophagitis		1829.5 (246.7)
	non-severe esophagitis		2218.6 (1042.6)
Sabban, 2021 [34] (median, IQR)	GER with respiratory symptoms	47.0 (37.0–54.0)	2461.0 (2129.0–2887.0)
	GER with digestive symptoms	50.0 (40.6–70.6)	2183.0 (1780.5–2527.5)
Di Chio, 2019 [35] (mean, SD)	GERD	26.4 ± 18.9	834.3 ± 473.3
	without evidence of GERD	48.4 ± 22.1	2608.2 ± 830.9

AET, acid exposure time; GER, gastroesophageal reflux; GERD, gastroesophageal reflux disease; NERD, non-erosive reflux disease; MNBI, mean nocturnal baseline impedance; PSPW, post-reflux swallow-induced peristaltic wave; FH, functional heartburn; RH, reflux hypersensitivity; AET, acid exposure time; SD, standard deviation; IQR, interquartile range.

**Table 2:** Overview of the key characteristics found in the eligible studies.

Author, Publication Year	Period of Time	Number of Patients	Age: Mean ± SD or Interval	Number of Pediatric Centers	Location	Endoscopy/Histology	MII Duration (Hours)	Statistical Result	Clinical Results (Study Conclusion)
Blasi, 2023 [29]	Jan. 2014–Apr. 2019	68	5–17 years	6	Italy	yes/yes	NM	- MNBI values were lower in NERD and in normal AET patients (statistically significant only between normal AET and RH and FH) - PSPW index was lower in NERD children compared to other phenotypes, with no statistical difference among NEEPs	- a lower distal MNBI was noted in NERD and normal AET compared to RH and FH; this result aligns with the presence of (microscopic) inflammation, reflux-induced impairment of mucosal integrity, and acid exposure in NERD - a lower PSPW index was observed in NERD children compared to other phenotypes; the PSPW index was reported as being able to discriminate between GERD and non-GERD adult subjects, as well as between NERD and FH
Eiamkulbutr, 2023 [25]	Feb. 2019–Dec. 2022	51	2.24 (1.11–7.67) years	1	Thailand	yes/yes	22.09 (20.20–2.41)	- in the multivariable analysis, MNBI was the independent parameter that was significantly different in participants diagnosed with GERD - MNBI yielded an area under the curve of 0.726 (95%CI: 0.581–0.870) - a cutoff value of 1466 ohm for MNBI had a sensitivity of 50.0% and a specificity of 33.33%	- MNBI is a novel parameter that should be integrated into the GERD diagnostic criteria in children
Tortoriello, 2023 [30]	NM	32	NM	2	Mexico	NM	NM	- higher values of PSPW index and MNBI were reported in functional disorders compared to patients with conclusive GERD - an inverse association was reported between MNBI, the PSPW index, and the acid exposure time	- the MNBI and PSPW index are novel markers that may be useful in the detection and categorization of patients with the clinical spectrum phenotype of GERD and in the differentiation from other functional gastrointestinal disorders in the pediatric population
Rosado-Arias, 2022 [31]	2015–2020	68	45 (2–216) months	1	Mexico	yes/yes	22 (16–33)	- MNBI at channel 6 was lower in patients with pathological AET than in those with normal AET	- children with pathological AET had lower impedance values of MNBI than those with normal AET - MNBI measurements should be part of the routine impedance pH assessment in children
Sabban, 2021 [32]	Jan. 2017–Nov. 2020	60	11.15 (5–17) years	1	Argentina	yes/yes	NM	- PSPW index and MNBI showed statistically significant lower values in patients with the RH phenotype compared to those with FH	- PSPW and MNBI are parameters that could enhance understanding of the esophageal behavior in patients with different reflux phenotypes
Rosado-Arias, 2021 [33]	2015–2020	68	45 (2–216) months	1	Mexico	yes/NM	NM	- in children with severe esophagitis, the MNBI was lower than in patients with non-severe esophagitis	- the measurement of impedance could prevent the need for upper gastrointestinal endoscopy for the diagnosis of esophagitis
Sabban, 2021 [34]	May 2018–Apr. 2021	92	8 (6.0–11.2) years	2	Argentina	NM	NM	- PSPW index was higher in the group with gastrointestinal symptoms - MNBI was higher in the group with respiratory symptoms	- children had different PSPW index and MNBI values according to the type of symptoms they had; these findings could orient clinical management
Di Chio, 2019 [35]	NM	40	7.9 ± 4.2 years	1	NM	yes/yes	NM	- PSPW index was significantly lower in those with GERD diagnosis	- both MNBI and the PSPW index showed very promising results and were lower in patients with a GERD diagnosis
**Total/mean ± SD/n (%)**	**2014–2022**	**479**	**2 months–17 years**	**12**	**Europe, Asia, North America, South America**				

NM, not mentioned; SD, standard deviation; NEEPs, non-erosive esophageal phenotypes; NERD, non-erosive reflux disease; MNBI, mean nocturnal baseline impedance; PSPW, post-reflux swallow-induced peristaltic wave; FH, functional heartburn; RH, reflux hypersensitivity; AET, acid exposure time.

**Table 3:** Impedance parameters (PSPW index, MNBI) for the main gastroesophageal reflux phenotypes.

Gastroesophageal Reflux Phenotype	Study	PSPW Index %	MNBI (ohms)
GERD	Tortoriello, 2023 [30] (mean ± SD)	20.11 ± 28.84	1673.11 ± 598.67
	Di Chio, 2019 [35] (mean ± SD)	26.4 ± 18.9	834.3 ± 473.3
	**Overall**	**23.25 ± 23.87**	**1253.7 ± 535.98**
NERD	Blasi, 2023 [29] (median, IQR)	42.6 (29.6–45.8)	1315 (1018–2832)
RH	Blasi, 2023 [29] (median, IQR)	56.3 (38.7–67.2)	2724 (2273–3403)
	Tortoriello, 2023 [30] (mean ± SD)	47 ± 29.23	2303 ± 837.8
	Sabban, 2021 [32] (mean ± SD)	48.4 ± 20.84	1286 ± 514.68
	**Overall**	**47.7 ± 25.03**	**1794.5 ± 676.24**
FH	Blasi, 2023 [29] (median, IQR)	52 (35.9–69.1)	2576 (2115–3014)
	Tortoriello, 2023 [30] (mean ± SD)	52 ± 43.85	2417 ± 656.23
	Sabban, 2021 [32] (mean ± SD)	64.6 ± 38.84	2168 ± 449.03
	**Overall**	**58.3 ± 41.34**	**2292.5 ± 552.63**

GERD, gastroesophageal reflux disease; NERD, non-erosive reflux disease; FH, functional heartburn; RH, reflux hypersensitivity; PSPW index, post-reflux swallow-induced peristaltic wave; MNBI, mean nocturnal baseline impedance, SD, standard deviation; IQR, interquartile range.

## Data Availability

The data presented in this study are available upon request from the corresponding author.
